# Malignant melanoma in a 12‐year‐old boy 17 months after completing hepatoblastoma treatment

**DOI:** 10.1002/cnr2.2118

**Published:** 2024-05-27

**Authors:** Koji Kanezawa, Hiroshi Yagasaki, Ayumu Arakawa, Reina Hoshi, Shuichiro Uehara, Ichiro Morioka

**Affiliations:** ^1^ Pediatrics Nihon University Itabashi Hospital Tokyo Japan; ^2^ Department of Pediatric Oncology National Cancer Center Hospital Tokyo Japan; ^3^ Pediatric Surgery Nihon University Itabashi Hospital Tokyo Japan

**Keywords:** chemotherapy, hepatoblastoma, melanoma, secondary malignant neoplasm

## Abstract

**Background:**

Melanoma is rare as a secondary malignant neoplasm among childhood cancer survivors.

**Case:**

We report a case of a 12‐year‐old boy who developed malignant melanoma with systemic metastases 17 months after completing treatment for hepatoblastoma. The diagnosis was made unexpectedly based on a bone marrow examination. The patient did not respond to immune checkpoint inhibitor therapy and died 6 weeks after being diagnosed with melanoma. Whole‐exome sequencing to examine 103 genes associated with cancer predisposition did not identify any germ‐line variants.

**Conclusion:**

This case study provides a unique example of melanoma in a childhood cancer survivor following hepatoblastoma treatment but does not identify any candidate variant to link hepatoblastoma and melanoma.

## 1 INTRODUCTION

Among the late sequelae after childhood cancer treatment, secondary malignant neoplasm (SMN) has an incidence of 2.6%.^1^ SMN significantly reduces the long‐term survival of childhood cancer survivors (CCS).^2^ Hematological malignancies generally occur within 10 years after treatment completion, whereas solid tumors have a later onset.^3^ Of these, secondary melanomas are rare, occurring in 0.14% of all CCS.^1^ Available data on SMN after treatment for hepatoblastoma is limited. A recent Japanese prospective study reported SMN in 13 out of 300 long‐term survivors who received treatment for hepatoblastoma. Of these, 11 were hematologic malignancies, including 9 cases of leukemia and one case each of lymphoma and myelodysplastic syndrome.^4^ Only two patients developed solid malignant tumors, namely Ewing sarcoma and thyroid cancer. Herein, we present a unique case of a Japanese boy who developed melanoma only 17 months after completing treatment for hepatoblastoma. Additionally, we analyzed the patient's genetic background coinciding with two oncologic events.

## 2 CASE

A 10‐year‐old boy was referred to Nihon University Itabashi Hospital for persistent fever and a large abdominal mass. His medical and family history were unremarkable. Abdominal computed tomography (CT) revealed a large tumor (16 cm) and multiple nodules in the lungs. Serum alpha‐fetoprotein (AFP) levels were elevated (31 290 mg/L; reference level < 10 mg/L). The patient was diagnosed with histologically confirmed embryonal‐type hepatoblastoma. The clinical stage was PRETEXT III with distant metastasis. Before surgery, he received three courses of the vincristine + irinotecan (VI) regimen and three courses of the cisplatin + 5‐fluorouracil + vincristine + doxorubicin (C5VD) regimen. As shown in Figure S1, the primary lesion shrank, the pulmonary metastases disappeared, and the AFP level decreased to within the reference range. Subsequently, extended right lobectomy of the liver was performed. He was treated with a further course of VI and three courses of C5VD as adjuvant chemotherapy and followed up with monthly lung and abdominal CT.

**FIGURE 1:**
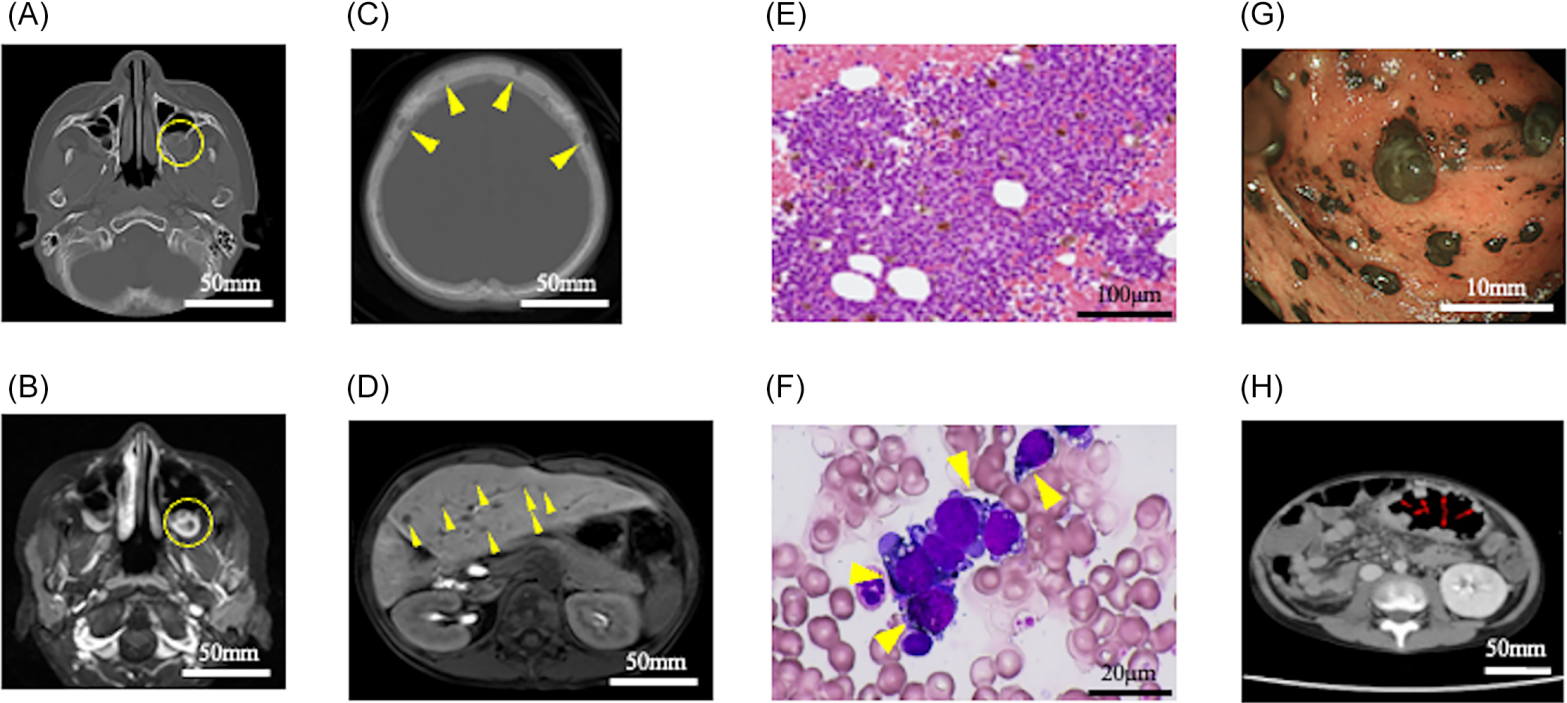
Imaging studies at the onset of melanoma. Panels (A) (enhanced brain computed tomography [CT]) and (B) (enhanced brain magnetic resonance imaging [MRI]) show osteolytic tumors in the left maxillary (yellow circles). Panel (C) (enhanced brain MRI) shows multiple osteolytic lesions in the skull (yellow arrows). Panel (D) (enhanced abdominal MRI) shows multiple nodules in the liver (yellow arrows). Panel (E) (clot section of the bone marrow, hematoxylin–eosin staining, ×400) shows massive invasion of tumor cells in the marrow. Panel (F) (bone marrow film, May–Giemsa staining, ×1000) shows the proliferation of aberrant cells with black granules, vacuoles, and blebs in the marrow, which replaced 70% of the bone marrow (yellow arrows). Panels (G) (endoscopic findings of the colon) and (H) (enhanced CT of the colon) show the mucosal invasion of melanoma cells in the colon (red arrows).

Seventeen months after completing the treatment, the patient developed progressive headaches and vomiting. Imaging studies revealed multiple osteolytic lesions in the skull and a small nodular lesion adhering to the left maxillary sinus (Figure 1A–C). In addition, abdominal CT revealed multiple nodules in the left lobe of the liver (Figure 1D), suggesting local hepatoblastoma recurrence with brain/skull metastases. However, the AFP level remained within the normal range (2.1 mg/L). He had mild thrombocytopenia (white blood cell count: 4.0 × 10^9^/L; hemoglobin level, 116 g/L; platelet count, 114 × 10^9^/L), leading us to suspect secondary leukemia. A bone marrow biopsy revealed aberrant cell characteristics with black granules, vacuoles, and blebs in 70% of the marrow cells (Figure 1E, F). Flow cytometry showed positive results for CD10 (91%) and negative results for other hematopoietic lineage markers (Figure S2). Moreover, complex chromosomal abnormalities (51~53, XY, i(1)(q10), i(6)(p10), +7, +9, −11, +add(12)(q?), +13, +20, and +mar) were detected in 10 of the 12 metaphases. Immunohistology was positive for S100, Melan‐A, and HMB‐45, leading to the diagnosis of melanoma with metastases to the liver, brain (skull), bone marrow, and colon (Figure 1G,H). Emergency chemotherapy was required for concurrent disseminated intravascular coagulation. Given that the patient was negative for the *BRAF* mutation, combination therapy was initiated with two courses of nivolumab (80 mg/course) and ipilimumab (1 mg/kg/course). However, the patient did not respond to immune checkpoint inhibitor therapy and died 6 weeks after admission. Thereafter, programmed cell death ligand‐1 (PDL‐1) was histologically confirmed to be negative in the melanoma cells.

**FIGURE 2:**
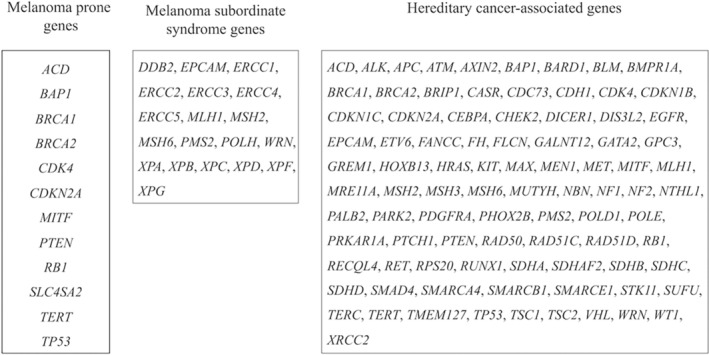
Target genes used in the analysis. Based on an updated literature review, we selected 103 genes as candidates for cancer predisposition in the patient.

Given that the patient had developed two rare cancers with a short latency, we presumed that he had a genetic predisposition to cancer. Based on recent review articles, we focused 103 genes closely associated with melanoma or hereditary cancer, as shown in Figure 2.^5^, ^6^ To find any candidate variants, we used whole‐exome sequencing (WES), which was performed by Riken Genesis Co., Ltd. (Tokyo, Japan). The patient's peripheral blood DNA samples were analyzed using the SureSelectXT Target Enrichment System for the Illumina Platform (Agilent Technologies). Genomic DNA was extracted with the Maxwell RSC Blood DNA Kit (Promega), sheared into approximately 200‐bp fragments, and used as a library for multiplexed paired‐end sequencing with the SureSelectXT Reagent Kit (Agilent Technologies). The prepared DNA libraries were hybridized to biotinylated cRNA oligonucleotide baits using the SureSelectXT Human All Exon V6 Kit (Agilent Technologies) for target enrichment. The hybridized DNAs were captured by magnetic beads and amplified. Target libraries were sequenced on an Illumina NovaSeq 6000 platform in a paired‐end 151‐bp configuration.

Adapter sequences were removed by cutadapt (ver. 1.2.1).^7^ After quality control, the reads were mapped to the reference human genome (hg19) using BWA (ver. 0.7.10).^8^ Mapping results were corrected using Picard (ver. 1.73, [http://broadinstitute.github.io/picard]) to remove duplicates and GATK (ver. 1.6–13) for local alignment and quality score recalibration.^9^ Single Nucleotide Variant (SNV) and Indel calls were analyzed with multi‐sample calling using GATK and filtered to coordinates with VQSR passed, and variant call quality score ≥ 30. SNVs and Indels were annotated based on dbSNP151, CCDS (NCBI, Release 15), RefSeq (UCSC Genome Browser, Feb 2018), Gencode (UCSC Genome Browser, ver. 19), and 1000Genomes (phase3 release v5). Variants were further filtered according to the following criteria: predicted functions of frameshift, nonsense, read‐through, missense, deletion, insertion, and insertion–deletion.

**FIGURE 3:**
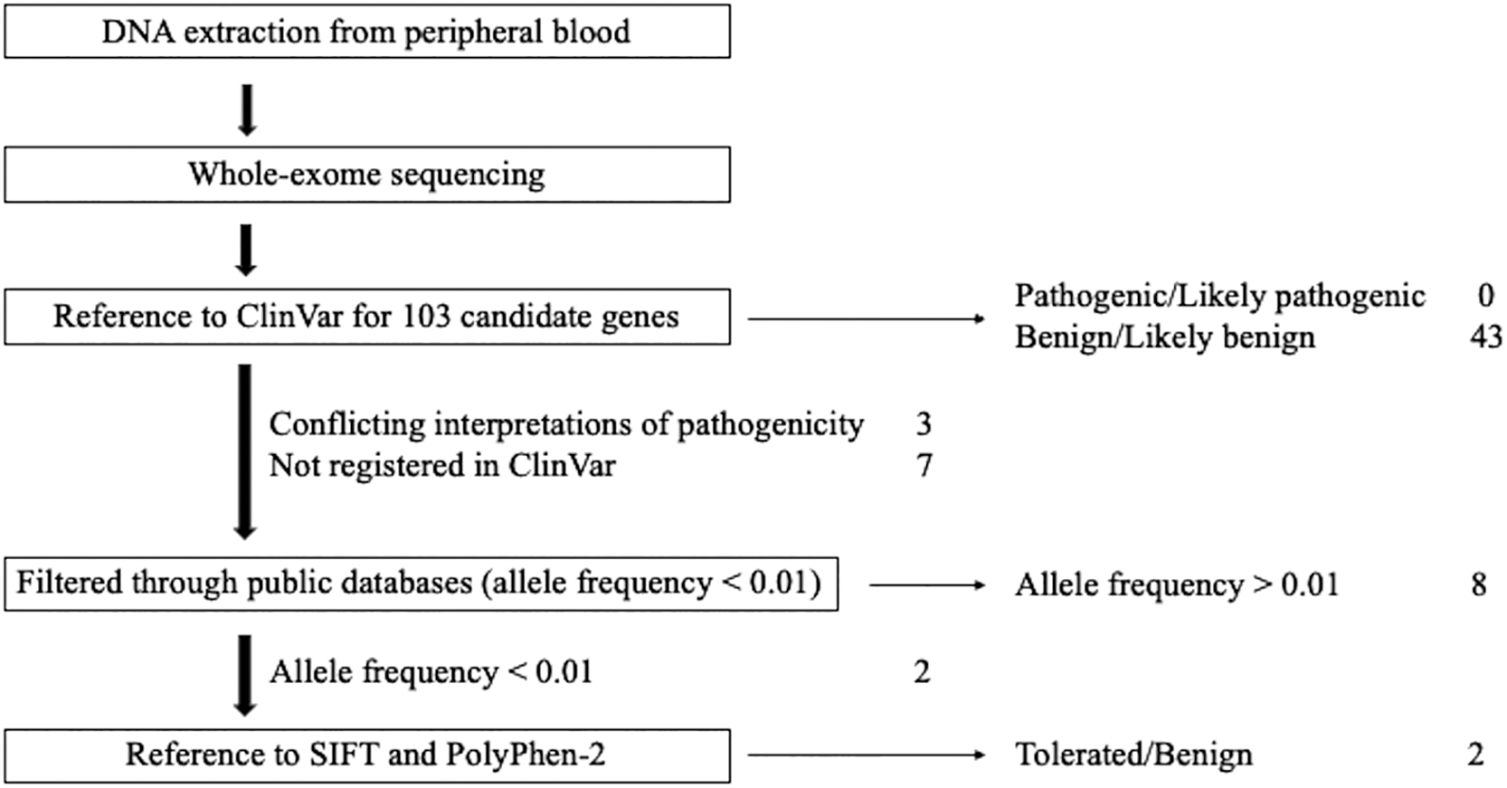
Genetic analysis pipeline and results. A total of 14 410 variants were extracted from the whole‐exome sequencing data. We evaluated the pathogenicity of 53 variants on the 103 melanoma or childhood cancer‐associated genes. Among the variants, 43 were benign or likely benign, and no pathogenic or likely pathogenic variants were identified based on the ClinVar database. Then, the other 10 variants were screened using variant allele frequency (VAF) in three major genome variant databases (1000Genomes, Human Genetic Variation Database, and The Genome Aggregation Database). The VAFs of 8 variants were greater than 1%, and the VAFs of the remaining 2 variants, CDKN2A (p. His66Arg) and PTCH1 (p. Arg1442Gln) were less than 1%. We analyzed the function of these rare variants using in silico prediction software (Sorting Intolerant From Tolerant and Polymorphism Phenotyping v2). Both variants were classified as tolerated and benign.

The analysis pipeline and results are summarized in Figure 3. In total, 14 410 variants were extracted from the whole‐exome sequencing data. Among these variants, 53 were identified in the 103 genes listed in Figure 2. Of these, 43 were benign or likely benign, and no pathogenic or probably pathogenic variants were identified based on the ClinVar database (https://www.ncbi.nlm.nih.gov/clinvar/) (Table S1). Then, the other 10 variants were screened using variant allele frequency (VAF) in three major genome variant databases (1000Genomes, Human Genetic Variation Database, and The Genome Aggregation Database). The VAF was more than 0.01 (1%) in eight variants, and less than 0.01 in the other two variants: CDKN2A (p. His66Arg) and PTCH1 (p.Arg1442Gln). The ClinVar database reported them as “Conflicting interpretation of pathogenicity.” Therefore, we evaluated the significance of these variants using in silico prediction software (Sorting Intolerant From Tolerant [https://sift.bii.a-star.edu.sg] and Polymorphism Phenotyping v2 [http://genetics.bwh.harvard.edu/pph2/index.shtml]). The results indicated that both variants were “tolerated and benign.”

## 3 DISCUSSION

This report describes a 12‐year‐old boy who developed melanoma following treatment of hepatoblastoma. No suggestive germline alterations were identified. However, this study is unique and novel since no similar genetic studies have been reported regarding a solid malignant neoplasm after hepatoblastoma.

Several reviews have reported that approximately 10% of melanomas occur in patients with a family history or germline alterations in melanoma‐prone genes (Figure 2).^5^, ^10^ Melanoma as an SMN is strongly associated with primary childhood neoplasms such as hereditary retinoblastoma, Hodgkin's lymphoma, soft tissue sarcoma, and germ cell cancer.^1^, ^5^ Radiotherapy for the primary neoplasm is also a risk factor for melanoma.^11^ However, this patient did not have any of these risk factors.

Hepatoblastoma tends to occur in infancy and sporadically. No predisposing genes for hepatoblastoma have been identified, but Beckwith–Wiedemann syndrome, trisomy 18, and familial adenomatous polyposis are associated with an increased risk of hepatoblastoma.^12^ This patient did not have any of these clinical conditions.

Generally, the latency from primary childhood cancer to secondary solid tumor is 10 years or longer.^3^, ^10^ Therefore, in this case, both the age of onset of hepatoblastoma and the short latency period from hepatoblastoma to melanoma diagnosis are atypical.

The complex chromosomal change in this patient was assumed to be induced by long‐term and intensive chemotherapy, termed chromoplexy or multifocal genomic crisis.^13^ Notably, similar complex chromosomal changes found in this patient's bone marrow have been reported in other patients with melanoma.^14^ We speculated that chromoplexy might have transformed visceral melanocytes into melanoma.

Melanomas are resistant to conventional cytotoxic chemotherapy. Molecular targeted drugs (BRAF or MEK inhibitors) and immune checkpoint inhibitors are available in Japan. According to a recent analysis, the objective response rate to combination therapy with nivolumab and ipilimumab is 58% in adult patients with advanced melanoma, and the median progression‐free survival is 11.5 months.^15^ The effectiveness of the immune checkpoint inhibitors depends on the PD‐1/PDL‐1 interaction between T cells and the tumor.^16^ Therefore, we inferred that this patient did not respond to the immunotherapy because PDL‐1 was not expressed on the melanoma cells.

This case study has some limitations. First, our analysis was based on whole‐exome sequencing, and a limited number of genes was evaluated. Whole‐genome sequencing (WGS) also may find unknown variants, however, their clinical significance is difficult to determine in a single patient. The accumulation of genome data through the genome profiling tests could be useful to evaluate WGS results. Second, we used the in silico software to predict the significance of two variants on CDKN2A and PTCH1. According to a recent study assessing the accuracy of prediction for four cancer‐risk genes, the sensitivity and specificity of SIFT were 99% and 56.4%, and those of Polyphen‐2 were 81%–90% and 55.6%–70.1%, respectively.^17^ The results indicate that the false‐negative rate is low, but the false‐positive rate is high. Therefore, the current result, “tolerated and benign,” in this case, is probably true. Conversely, the result “deleterious and damaging” should be interpreted with caution in the absence of additional functional evidence.

In conclusion, this report provides a unique example of melanoma in a CCS following hepatoblastoma treatment. Despite the screening analysis using WES, we could not find a candidate gene to link hepatoblastoma and melanoma.

## AUTHOR CONTRIBUTIONS

**Koji Kanezawa:** Conceptualization (equal); investigation (equal); methodology (equal); writing – original draft (lead). **Hiroshi Yagasaki:** Conceptualization (equal); methodology (equal); writing – review and editing (equal). **Ayumu Arakawa:** Investigation (equal). **Reina Hoshi:** Investigation (equal). **Shuichiro Uehara:** Investigation (equal). **Ichiro Morioka:** Writing – review and editing (equal).

## CONFLICT OF INTEREST STATEMENT

The authors have stated explicitly that there are no conflicts of interest in connection with this article.

## ETHICS STATEMENT

All procedures performed in this study involving human participants were in accordance with the ethical standards of the institutional and/or national research committee and the 1964 Declaration of Helsinki and its later amendments (or comparable ethical standards). This case study was approved by the institutional review board of Nihon University Itabashi Hospital (GM20‐21‐0).

## PATIENT CONSENT STATEMENT

The publication of this report was approved by the institutional review board of Nihon University Itabashi Hospital (GM20‐21‐0). We obtained the opt‐in consent from the patient's parents.

## Supporting information


**Figure S1.** Clinical course of hepatoblastoma in the patient. The upper panel shows the alpha‐fetoprotein (AFP) levels, and the lower panel shows the chemotherapy regimens. VI regimen: VCR (1.5 mg/m^2^ on days 1 and 8), Ir (50 mg/m^2^ on days 1–5). C5VD regimen: CDDP (100 mg/m^2^ on day 1), 5‐FU (600 mg/m^2^ on day 2), VCR (1.5 mg/m^2^ at days 2, 9, and 16), DXR (30 mg/m^2^ on days 1 and 2). 5‐FU, 5‐fluorouracil; AFP, alpha‐fetoprotein; CDDP, cisplatin; DXR, doxorubicin; Ir; irinotecan; VCR, vincristine.

**Figure S2.** Flow cytometric analysis of bone marrow cells. Two‐dimensional analysis of CD45 and SSC by flow cytometry (left figure) identifies three cell populations, R1, R2, and R3. The major population (R1) located in the CD45‐negative area expresses CD10 but not CD1a, 2, 3, 4, 5, 7, 8, 13, 14, 19, 20, 28, 34, 41, 56, 117, Glycophorin A, or PD‐1. The results indicate that R1 is a non‐hematological tumor cell infiltrating the bone marrow, whereas the minor populations, R2 and R3, are located in the CD45‐positive area and represent normal myeloid precursors (positive; CD13, 33 and 117) and normal lymphocytes (data not shown), respectively. PD‐1, programed cell death‐1; SSC, Side Scatter.

**Table S1.** Characteristics of 53 variants identified by whole‐exome sequencing (WES).

## Data Availability

The data that support the findings of this study are available from the corresponding author upon reasonable request.
