# Supplementary figures and images for: Malignant melanoma in a 12‐year‐old boy 17 months after completing hepatoblastoma treatment
Source: Cancer Rep (Hoboken). 2024 May 27;7(5):e2118. doi: 10.1002/cnr2.2118 (PMC11129619; doi:10.1002/cnr2.2118)

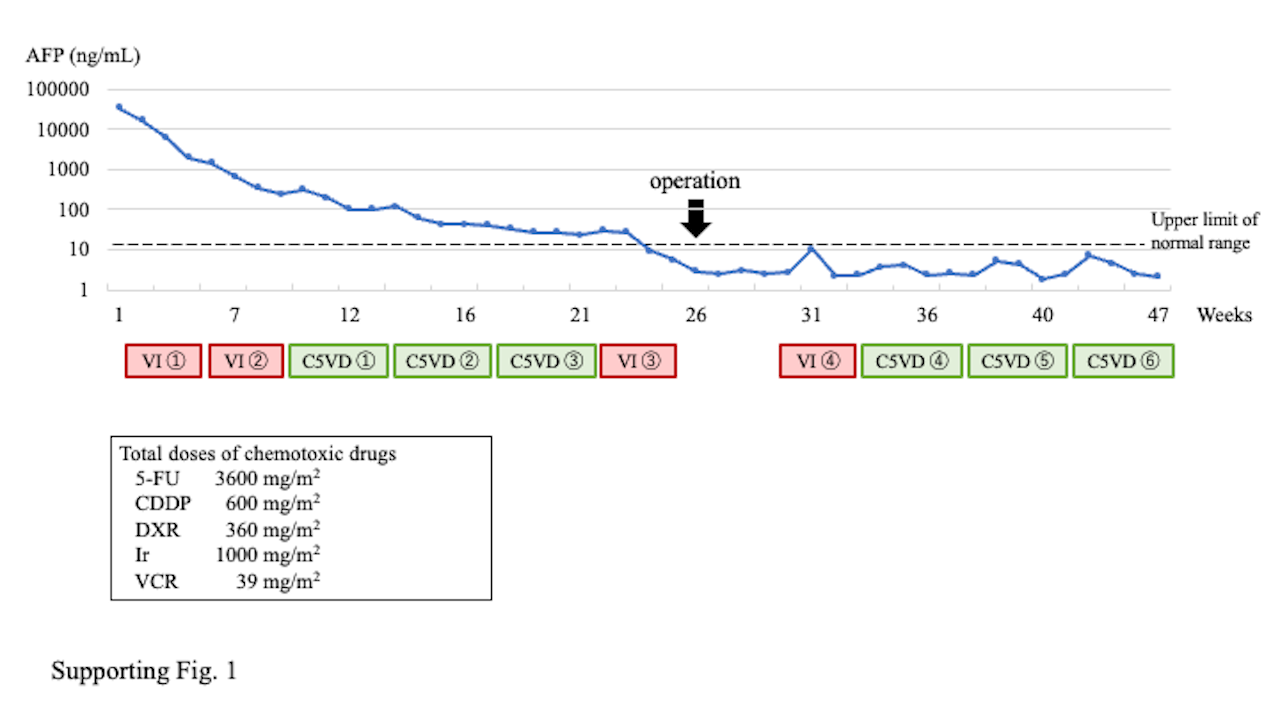

Supplement: Supplementary file 1 — Figure S1. Clinical course of hepatoblastoma in the patient. The upper panel shows the alpha‐fetoprotein (AFP) levels, and the lower panel shows the chemotherapy regimens. VI regimen: VCR (1.5 mg/m2 on days 1 and 8), Ir (50 mg/m2 on days 1–5). C5VD regimen: CDDP (100 mg/m2 on day 1), 5‐FU (600 mg/m2 on day 2), VCR (1.5 mg/m2 at days 2, 9, and 16), DXR (30 mg/m2 on days 1 and 2). 5‐FU, 5‐fluorouracil; AFP, alpha‐fetoprotein; CDDP, cisplatin; DXR, doxorubicin; Ir; irinotecan; VCR, vincristine. [file CNR2-7-e2118-s002.tiff]
